# Retraction Note to: Universal minicircle sequence binding protein of *Leishmania donovani* regulates pathogenicity by controlling expression of cytochrome-b

**DOI:** 10.1186/s13578-022-00875-7

**Published:** 2022-08-22

**Authors:** Ruby Singh, Bidyut Purkait, Kumar Abhishek, Savita Saini, Sushmita Das, Sudha Verma, Abhishek Mandal, Ayan Kr. Ghosh, Yousuf Ansari, Ashish Kumar, Abul H. Sardar, Ajay Kumar, Pradeep Parrack, Pradeep Das

**Affiliations:** 1 Department of Molecular Parasitology and Bioinformatics, Rajendra Memorial Research Institute of Medical Sciences (RMRIMS), Indian Council of Medical Research (ICMR), Agamkuan, Patna, Bihar 800007 India; 2 Department of Biotechnology, National Institute of Pharmaceutical Education and Research (NIPER), Vaishali, Hajipur, Bihar 844101 India; 3 Department of Microbiology, All India Institute of Medical Sciences (AIIMS), Patna, 801105 India; 4 Department of Biochemistry, Bose Institute, Kolkata, 700009 India

## Retraction to: Cell Biosci (2016) 6:13 10.1186/s13578-016-0072-z

The Editors-in-Chief have retracted this article. After publication, concerns were raised regarding the data presented in Figs. 4–6 and S4. Specifically:

- Figs. 4i and 5f appear to contain overlap;
- Figs. 4e and 6i appear to contain overlap;
- Figs. 4h and 6a appear to share the same control bands (stretched/squished);
- There are similarities in various groups in Fig. 6e;
- Figs. S4a and S4c appear to contain the same gel with different exposure (as a mirror image).

The authors have been unable to provide the full raw data to address these concerns. The Editors-in-Chief therefore no longer have confidence in the presented data.

Ruby Singh does not agree to this retraction. Yousuf Ansari and Pradeep Das have not explicitly stated whether they agree to this retraction notice. Bidyut Purkait, Kumar Abhishek, Savita Saini, Sushmita Das, Sudha Verma, Abhishek Mandal, Ayan Kr. Ghosh, Ashish Kumar, Abul H. Sardar, Ajay Kumar and Pradeep Parrack have not responded to any correspondence from the editor or publisher about this retraction.

